# Subcuticular Barbed Suture and Skin Glue Wound Closure Decreases Reoperation and Length of Stay in Geriatric Hip Fractures When Compared With Staples

**DOI:** 10.5435/JAAOSGlobal-D-21-00205

**Published:** 2021-10-04

**Authors:** Emily Zhao, Ivan S. Tarkin, Gele B. Moloney

**Affiliations:** From the Department of Orthopaedic Surgery, University of Pittsburgh Medical Center, Pittsburgh, PA.

## Abstract

Patients with geriatric hip fracture are notoriously frail and at risk for complications. Persistent postoperative wound drainage can lead to prolonged hospital stay, increased risk for infection, and need for revision surgery. The purpose of this study was to determine the effect of wound closure technique, barbed monofilament subcuticular suture and skin glue versus staples on rates of intervention for wound drainage and length of hospital stay after geriatric hip fracture fixation.

**Methods:**

A retrospective review of isolated hip fractures in patients older than 60 years at a single institution over a 3-year period was done. Hip fractures included femoral neck, intertrochanteric, and subtrochanteric femur fractures treated with internal fixation or arthroplasty. Skin closure technique, at the discretion of the operating surgeon, included either barbed subcuticular monofilament suture and skin glue or staples. Charts and radiographs were reviewed to determine patient characteristics, Charlson Comorbidity Index, type of wound closure, length of stay, and interventions for persistent wound drainage.

**Results:**

There were 175 patients in the barbed suture and skin glue group and 211 patients in the staples group. The barbed suture group had an average postsurgical length of stay of 5.0 days which was significantly lower than the staples group (7.0 days, *P* < 0.00001). In the staples group, 17 patients (8%) required incisional negative pressure wound therapy due to wound drainage with five patients (2.4%) returning to the operating room secondary to persistent wound drainage. No patients were observed in the barbed suture group that required intervention for wound drainage.

**Discussion:**

Barbed suture and skin glue closure is associated with markedly shorter hospital stay and fewer interventions for wound drainage when compared with staples after surgical treatment of geriatric hip fractures.

Hip fractures are among the most common and morbid fragility fractures in the elderly population. The incidence in the United States in 2005 was 793.5 per 100,000 in women and 369 per 100,000 in men.^1^ One-year mortality is commonly reported at 30% after geriatric hip fractures^2,3^; however, a recent review of worldwide registry data shows an improvement in 1-year mortality to 22%,^4^ with data from the United States showing a rate of 21%.^5^ A review of Medicare data from 2014 showed that new hip fractures in patients older than 65 years cost the US healthcare system about $5.96 billion, with the majority of the per patient cost attributed to the inpatient hospitalization and skilled nursing facilities.^6^

Inpatient length of stay (LOS) after geriatric hip fracture can be variable across the nation, likely dependent on patient demographics and hospital protocols. The average LOS has been reported from 4.6^7^ to 12 days^8^; however, lengthier hospitalizations can occur. Systemic initiatives, such as decreasing the time to surgery for treatment of geriatric hip fractures, can help decrease the overall LOS and minimize the risk of complications.^9,10,11,12^ Increased medical comorbidities may increase perioperative complications,^13^ such as respiratory infections, bleeding requiring transfusions, venous thromboembolism, cardiac failure, and delirium,^14,15^ which may also increase LOS.^16^ Although many of these factors are out of the operating surgeon's control, some factors such as implant choice, technical ability, and wound management are within the surgeon's purview. Utilization of surgical techniques that can decrease the risk of prolonged LOS can help improve outcomes for patients with hip fracture. Inadequate wound closure may lengthen hospital stay and increase costs if there is need for an intervention to improve wound healing.

Skin staple closure is commonly used in both hip arthroplasty and hip fracture surgery. Barbed subcuticular closure has been investigated with some evidence to support its use in the elective total hip arthroplasty population.^17,18^ Barbed suture has been shown to be biomechanically comparable with monofilament suture secured with knots^19^ and able to hold a more “watertight” seal at the surgical incision,^20^ which in theory can help decrease postoperative wound discharge that has been linked to surgical site infection.^21^ Skin glue, 2-octyl cyanoacrylate, has also been associated with decreased postoperative wound drainage in the fracture setting.^22,23^ The geriatric fracture population presents a unique set of challenges because patients often present with decreased tissue quality, malnutrition,^24^ and limited mobility, which may raise the likelihood of wound-related complications compared with the elective population. In addition, these patients are notoriously frail, and any complication has the potential to be catastrophic.

To the best of our knowledge, there are no studies examining the use of barbed subcuticular suture or skin glue versus skin staples on the rate of wound drainage in geriatric hip fracture treatment. At our two level 1 trauma tertiary referral centers, geriatric hip fractures are routinely closed with either skin staples or barbed subcuticular suture and skin glue based on surgeon preference. We hypothesize that barbed suture and glue allows for better wound management by decreasing wound ooze and need for subsequent intervention to address wound-related issues. In addition, we sought to determine the effect of wound closure on LOS.

## Methods

After Institutional Review Board approval, a retrospective review of isolated hip fractures in patients older than 60 years treated by three fellowship-trained orthopaedic traumatologists at our institution from January 2017 through November 2019 was done. Hip fractures were defined as Arbeitsgemeinschaft fur Osteosynthesefragen/Orthopaedic Trauma Association (AO/OTA) 31 and 32, including femoral neck, intertrochanteric, and subtrochanteric femur fractures treated with internal fixation or arthroplasty. Internal fixation techniques include percutaneous cannulated screws, dynamic hip screws, and cephalomedullary nails. Arthroplasty techniques include cemented and noncemented hemiarthroplasty, total hip arthroplasty, and resection arthroplasty. Both level 1 tertiary referral centers where these surgeries were done are similar academic facilities where residents and fellows from the same training program are actively involved, including wound closure. The surgical technique between all three surgeons for the various procedures is very similar. Two of the three surgeons work at both hospitals where one works primarily at one site. Although there are nonorthopaedic trauma fellowship-trained surgeons taking call for emergencies at both hospitals, the overwhelming majority of hip fractures were taken care of by the three surgeons whose patients we have reviewed here and represent an appropriate diversity of simple to complex cases.

Patients were retrospectively divided into two groups based on the wound closure technique. Both groups underwent standard closure of deep and dermal layers with interrupted absorbable sutures. Skin closure was at the discretion of the treating surgeon and done using running subcuticular V-Loc 90, a barbed absorbable monofilament suture (Medtronic), and skin glue 2-octyl cyanoacrylate (Exofin high viscosity tissue adhesive, Chemence Medical) (barbed suture group), which was the preference of one surgeon, or standard metallic skin staples (staples group), the preference of the other two surgeons.

Charts, radiographs, and hospital billing records were reviewed to determine patient characteristics, Charlson Comorbidity Index (CCI), type of wound closure, LOS, and interventions for wound-related complications. Interventions included application of incisional negative pressure wound therapy (NPWT) and return to the operating room to address wound issues. For all three surgeons, the standard postoperative protocol regarding surgical wounds is to apply a dry dressing at the time of surgery with daily dry dressing changes starting on postoperative day 2. If there is minimal or no discharge on the dressing, generally the patient is considered appropriate for discharge from a wound perspective. If there is persistent postoperative wound ooze that is increasing or not decreasing, then typically NPWT is applied over the closed wound. This technique has been previously described for the management of prolonged postoperative wound drainage^25^ and remains in place for an average of 2 to 4 days depending on the output. After NPWT is removed, the wound is reevaluated. Débridement is done at the discretion of the treating surgeon because of either persistent wound ooze despite NPWT or overt evidence of wound infection.

Statistical analyses were done using the Student *t*-test for continuous variables and chi-squared tests for categorical variables. The Fisher exact test was used to compare revision surgery rates. Statistical significance was set at *P* < 0.05.

## Results

From January 2017 through November 2019, there were 441 isolated hip fracture cases treated at our institution by three fellowship-trained orthopaedic traumatologists. Fifty-five of these were excluded because the method of closure could not be determined based on chart review and imaging. This resulted in 386 eligible cases, 175 cases (174 individuals) that were closed with barbed suture and skin glue and 211 cases (209 individuals) that were closed with staples. The average age of the cohort was 79.0 ± 9.9 years. Two hundred forty-seven patients (64%) were female. No significant differences were observed between the barbed suture and staples groups for mean age, sex, body mass index (BMI), CCI, or time to initial surgery (Table 1). The overall 30-day mortality was 2.3% (4 patients) for the barbed suture group and 5.2% (11 patients) for the staples group (*P* = 0.13), which includes one of the 17 patients who required an intervention for wound-related complications.

**Table 1:** Comparison of Group Demographics, Surgical Techniques, Hospital LOS, and Complication Rates in Patients Undergoing Barbed Suture Closure Versus Staple Closure

	Barbed Suture (n = 175)	Staples (n = 211)	*P* Value
Age	78.5 ± 10.0	79.5 ± 9.8	0.36
% Female	69.1 (n = 121)	59.7 (n = 126)	0.06
BMI (kg/m^2^)	26.1 ± 6.6	25.8 ± 5.8	0.64
CCI	6.2 ± 2.8	6.3 ± 3.0	0.84
Time to initial surgery (d)	0.9 ± 1.1	1.0 ± 1.4	0.37
Open reduction and internal fixation	34 (19.4%)	18 (8.5%)	
Arthroplasty	38 (21.7%)	45 (21.3%)	
Cephalomedullary nail	101 (57.7%)	147 (69.7%)	
Resection	2 (1.1%)	1 (0.5%)	
Total LOS (d)	6.0 ± 3.0	8.1 ± 5.4	<0.00001
Postoperative LOS (d)	5.0 ± 2.7	7.0 ± 5.0	<0.00001
Intervention for wound-related complication	0 (0%)	17 (8.0%)	<0.001
Revision surgery for wound drainage	0	5 (2.4%)	0.09
30 d mortality rate	2.3 (n = 4)	5.2 (n = 11)	0.13

BMI = body mass index, CCI = Charlson Comorbidity Index, LOS = length of stay

### Wound-related Intervention

No patients within the barbed suture group required an intervention for wound-related complication. Seventeen patients in the staples group (8.0%) required incisional NPWT (*P* < 0.001), and five patients within that group (2.4% of overall stapled) required a return to the operating room for wound débridement (*P* = 0.09) (Table 1). Of the 17 patients who required NPWT, 10 underwent cephalomedullary nailing and 7 had arthroplasty procedures (Table 2), representing 15% of the arthroplasty staples group and 6.8% of the cephalomedullary nailing staples group. The mean number of days from surgery to placement of incisional NPWT was 5.3 ± 2.8 days, with mean 2.75 days of therapy in the 12 patients who did not require a return to the operating room. Of the five patients who required another operation for wound débridement or revision, the mean number of days from initial surgery to revision surgery was 25.4 ± 16.5 days. Three of these patients underwent cephalomedullary nailing and two had arthroplasty procedures. One of these patients died within 30 days because of reasons not related to their surgery, and another died within 60 days after undergoing hospice care with continuous incisional NPWT until death. One returned to the operating room at 2 months postoperatively for drainage and subsequently healed, and two had multiple reoperations because of drainage and presumed deep infection. Both of these last two patients had been healing with local wound care at their most recent follow-ups.

**Table 2:** Comparison of Demographics and LOS for Stapled Patients Subgroups

	Staples: Intervention Subgroup (n = 17)	Staples: No Intervention Subgroup (n = 194)	*P* Value
Age	78.7 ± 8.8	79.5 ± 9.8	0.74
% Female	47.0 (n = 8)	60.8 (n = 118)	0.27
BMI (kg/m^2^)	26.9 ± 5.3	25.6 ± 5.8	0.39
CCI	6.6 ± 2.7	6.3 ± 3.0	0.67
Time to initial surgery (days)	0.8 ± 0.6	1.1 ± 1.4	0.13
Total LOS (days)	13.0 ± 8.3^a^	7.7 ± 4.8^a^	<0.05
Postoperative LOS (days)	12.2 ± 8.4^a^	6.5 ± 4.3^a^	<0.05
Open reduction and internal fixation	0	18 (9.3%)	
Arthroplasty	7 (41.2%)	38 (19.6%)	
Cephalomedullary nail	10 (58.8%)	137 (70.6%)	
Resection	0	1 (0.5%)	
30 d mortality rate	5.9 (n = 1)	5.2 (n = 10)	0.89

BMI = body mass index, CCI = Charlson Comorbidity Index, LOS = length of stay

a Denotes statistically significant (*P* < 0.01) difference when compared with the barbed suture group.

Subgroup analysis compared patients with staples closure who underwent wound débridement with those who did not demonstrated no significant differences for age, BMI, or comorbidities between the two subgroups or compared with the barbed suture group (Table 2). The only notable differences between the two subgroups and compared with the barbed suture group were total and postoperative LOS.

### Length of Stay

Postoperative LOS was significantly shorter at 5.0 ± 2.7 days for the barbed suture group compared with 7.0 ± 5.0 days for the staples group, *P* < 0.0001. Overall LOS (8.1 ± 5.4 days in the staples group versus 6.0 ± 3.0 days in the barbed suture group, *P* < 0.00001) was also significantly shorter in the barbed suture group with no difference in average time to surgery (Table 1). When isolating the patients who required intervention for wound drainage, mean postoperative LOS increased substantially to 12.2 ± 8.4 days, almost 6 days greater than the no intervention staples group at 6.5 ± 4.3 days (*P* < 0.01) (Table 2).

## Discussion

When compared with staple closure, the use of barbed subcuticular suture and skin glue in the treatment of geriatric hip fractures results in a notable reduction in postoperative LOS and interventions for wound complications which supports our hypothesis. Although there were no differences between groups for age, BMI, comorbidities, and type of procedure done, LOS averaged 2 fewer days in the barbed suture and skin glue group. In addition, there were no interventions for wound-related complications in the barbed suture group compared with 8% of patients in the staples group who required additional interventions, including five patients (2.4%) who required return to the operating room.

LOS after hip fracture surgery is a large driver of cost in hip fracture care.^6^ In addition, an elevated LOS is associated with increased complication rates and 30-day mortality.^26^ In our series, barbed subcuticular suture and glue closure decreased both overall LOS and postoperative LOS by an average of 2 days. When excluding the 17 patients who required additional intervention, the average postoperative LOS in the remaining patients within the staples group was still markedly longer than the barbed suture group, whereas the age, BMI, and CCI of those patients remained similar between the two groups. Increased LOS, even in the absence of additional intervention, may be due to the need for additional inpatient monitoring of wound ooze. Persistent wound drainage after staple closure can be attributed to the inability of staple closure to create a watertight seal. A total hip arthroplasty study comparing barbed suture with staples found markedly longer time until the surgical wound was dry after staple closure.^17^ This persistent wound ooze delayed discharge similarly by an average of 2 days in certain patients. A British National Health System study on skin closure in hip and knee arthroplasty found a notable reduction in prolonged wound drainage and inpatient stay for wound exudate with the use of barbed suture and glue compared with staples or monofilament suture.^27^ They also conducted a cost-benefit analysis which demonstrated overall cost savings with the use of barbed suture, despite the more expensive cost of the suture compared with staples. The extra cost in using barbed suture was outweighed by its benefit in reducing the cost of prolonged inpatient hospitalization for wound observation.

A true cost analysis was not conducted in this study because of the limitations of our data collection. When examining material cost at our institution, the cost of the barbed subcuticular suture and skin glue was an additional $22 per patient compared with the cost of a skin stapler. Another consideration related to cost is surgical time. A study comparing nonbarbed subcuticular suture closure with staples in hip arthroplasty found that on average running, subcuticular suture closure added only 5 minutes to the surgical time.^28^ When comparing the cost of materials and surgical time for barbed suture and skin glue closure versus the cost of increased LOS, NPWT, and revision surgery in the staple closure group, a notable cost savings could certainly be implied.

To the best of our knowledge, this is the first study comparing the use of barbed suture and skin glue with staples in skin closure for hip fracture treatment. A randomized controlled trial looking at subcuticular Monocryl and skin glue compared with staple closure in acetabular surgery found a nonsignificant increase in revision surgery for wound complications in the staple group and a notable 1.5-day increase in days between surgery and a dry incision in the staple group.^22^ Although not evaluating barbed suture specifically, the addition of glue to a subcuticular closure may be beneficial in trauma patients.

This study is limited in that it was a retrospective analysis and we did not specifically examine reasons for prolonged postoperative LOS, such as exacerbations of medical comorbidities, delirium, bleeding requiring transfusions, and other possible postoperative complications. In addition, although anticoagulation could play a role in wound drainage, we did not specifically review what type of anticoagulation was given to those patients who needed an intervention for wound drainage versus those who did not. Typically, patients with geriatric hip fracture at our institutions are treated with low-molecular-weight heparin for thromboprophylaxis unless they are already on an anticoagulant at the time of admission. Given that both groups were closely matched for patient demographics and degree of comorbidities, we would not anticipate a notable difference in anticoagulation distribution between the two groups.

Even with the intervention group excluded, the nonintervention staples group still had a 1.5-day greater LOS compared with the barbed suture group, which was found to be notable. This difference in LOS could possibly be due to perioperative complications that we were unable to quantify, despite the similarity between the two cohorts. Another reason could be prolonged wound observance in the staples group that did not require any intervention. Although we cannot state that the increased LOS is attributable solely to the type of wound closure, the approximate 2-day difference is consistent with previous literature demonstrating a similar delay until postoperative wounds are dry after staple closure.^17,27^

Finally, this study examined several different types of fixations for proximal femur fractures which will have varying incisions and complexity of dissection. We are unable to retrospectively determine whether incision length itself was correlated with need for wound intervention. However, we did find that interventions for wound-related complications were more common in the arthroplasty group, with 15% of arthroplasty patients closed with staples requiring wound-related intervention compared with 6.8% of cephalomedullary nail patients closed with staples (Table 2). Despite the common conception that cephalomedullary nailing is a percutaneous operation, and therefore not high risk from a wound perspective, a clinically relevant portion of patients had wound-related complications in this cohort as well.

In conclusion, when compared with staples, we found that closure with subcuticular barbed suture and skin glue is effective in decreasing interventions required for prolonged wound drainage after geriatric hip fracture surgery and is associated with decreased postoperative LOS.
